# A comprehensive review of the literature on CD10: its function, clinical application, and prospects

**DOI:** 10.3389/fphar.2024.1336310

**Published:** 2024-02-08

**Authors:** Shudong Wang, Yinghui Xiao, Xingna An, Ling Luo, Kejian Gong, Dehai Yu

**Affiliations:** ^1^ Department of Cardiology, The First Hospital of Jilin University, Changchun, Jilin, China; ^2^ Public Research Platform, The First Hospital of Jilin University, Changchun, Jilin, China; ^3^ Department of Thoracic Surgery, The First Hospital of Jilin University, Changchun, Jilin, China

**Keywords:** CD10, cancer, obesity, type-2 diabetes, Alzheimer’s disease, cardiovascular disease, COVID-19, stem cells

## Abstract

CD10, a zinc-dependent metalloprotease found on the cell surface, plays a pivotal role in an array of physiological and pathological processes including cardiovascular regulation, immune function, fetal development, pain response, oncogenesis, and aging. Recognized as a biomarker for hematopoietic and tissue stem cells, CD10 has garnered attention for its prognostic potential in the progression of leukemia and various solid tumors. Recent studies underscore its regulatory significance and therapeutic promise in combating Alzheimer’s disease (AD), and it is noted for its protective role in preventing heart failure (HF), obesity, and type-2 diabetes. Furthermore, CD10/substance P interaction has also been shown to contribute to the pain signaling regulation and immunomodulation in diseases such as complex regional pain syndrome (CRPS) and osteoarthritis (OA). The emergence of COVID-19 has sparked interest in CD10’s involvement in the disease’s pathogenesis. Given its association with multiple disease states, CD10 is a prime therapeutic target; inhibitors targeting CD10 are now being advanced as therapeutic agents. This review compiles recent and earlier literature on CD10, elucidating its physicochemical attributes, tissue-specific expression, and molecular functions. Furthermore, it details the association of CD10 with various diseases and the clinical advancements of its inhibitors, providing a comprehensive overview of its growing significance in medical research.

## Introduction

CD10, also known as CALLA, CMT2T, SCA43, SFE, neutral endopeptidase, neprilysin (NEP), was first identified in 1975. This type II integral membrane protein is embedded within the plasma membrane, with its principal active site exposed to the extracellular environment. As an ectoenzyme, it facilitates the proteolytic breakdown of a variety of substrates (Maguer-Satta et al., 2011). The precise functions of CD10 across different species remain elusive. Initially regarded as a tumor-specific antigen, CD10 has also been found on numerous normal cell types, both haematopoietic and non-haematopoietic, yet its contribution to normal physiology and pathological states is not well defined. Research indicates that CD10 is not only pivotal in renal proteolysis, cardiovascular regulation, and immune response but also plays a role in cell proliferation, aging, and fetal development. Moreover, it is associated with a spectrum of pathological diseases, including Alzheimer’s disease (AD), diabetes, musculoskeletal diseases (such as osteoarthritis), cancer, cardiovascular diseases, and neurodegeneration (Greif et al., 2020; Nalivaeva et al., 2020). While there have been several reviews on CD10 (Nalivaeva et al., 2020; DElia et al., 2017; Feygina et al., 2019), the body of research from the past 5 years has unveiled additional biological roles for CD10, warranting an updated comprehensive review to deepen our understanding of this molecule. In this article, we aim to consolidate the most recent discoveries concerning CD10 and elucidate its involvement in the etiology and progression of diseases, as well as discuss the therapeutic potential of CD10 inhibitors in clinical settings.

## Biological roles of CD10

The human CD10 gene is located on chromosome 3q21-27, spanning over 80 kb and consisting of 24 exons. As a transmembrane protein, CD10 contains three regions: an extracellular region, a transmembrane region, and an intracellular region (DAdamio et al., 1989). The extracellular section of CD10 is pivotal for zinc binding and the catalytic activity (McKerrow, 1987). The molecular weight of the CD10 protein varies from 90 kDa to 110 kDa due to differing extents of glycosylation across tissues. The amino acid sequences of CD10 show about a 90% identity between human and rat, with 100% conservation of essential functional motifs (Malfroy et al., 1987). CD10 has been identified in various hematopoietic tissues, including human lymphoid progenitors, germinal center B-cells, neutrophils, and T-cells (Kalled et al., 1995; de Leval et al., 2001), as well as in nonhematopoietic tissues, such as the intestinal, breast, kidney, prostate, lung, liver, placenta, brain, gonads, adrenal gland, and neurons (Chu and Arber, 2000; Thong et al., 2014; Feygina et al., 2019). Moreover, CD10 enzymatic activity has been detected in bodily fluids like cerebrospinal fluid, plasma, and amniotic fluid (Spillantini et al., 1990).

CD10 serves as an integral enzyme across multiple organ systems, orchestrating critical biological processes such as deactivating enkephalins in the brain, neutralizing tachykinin in the airways, metabolizing cardiac bradykinin, and degrading neurotransmitter in nociceptive nerve fibers (Back and Gorenstein, 1989; Kramer et al., 2009; Manolis et al., 2020). As a ubiquitous zinc-metallopeptidase, CD10 is implicated in the regulation of numerous vital physiological pathways by modulating the bioactivity and availability of a plethora of peptides. The expression of CD10 serves to mediate cellular responses to hormones by fine-tuning local peptide concentrations. As an extracellular enzyme, CD10 facilitates the breakdown of various substrates, thus impacting the physiological and developmental functions of a wide range of tissues and organs through its action on peptides such as substance P, endothelin, members of the atrial natriuretic peptide family, somatostatin, adrenomedullin, glucagon, angiotensin I and II, and encephalin (Nalivaeva et al., 2020).

In the lungs, it is particularly abundant and crucial for cell differentiation, proliferation, and tumorigenesis (Shipp et al., 1991a). Sunday et al. found that bombesin-like peptides (BLP, a mitogen for normal bronchial epithelial cells and small cell lung carcinomas) was highly expressed in fetal lung tissue, and CD10 inhibition could strengthen the BLP-responsive small cell lung carcinomas (Sunday et al., 1992). Additionally, CD10’s presence in airways contributes to physiological and pathological responses by regulating inflammatory neuropeptides such as substance P, known for constricting airway smooth muscle (Borson, 1991).

Previous animal studies have found that CD10 presents in brain structures such as gray matter, caudate-putamen and choroid plexus (Matsas et al., 1986; Bourne and Kenny, 1990). Its distribution in the human brain is markedly heterogeneous, with concentration peaks in the globus pallidus and substantia nigra’s pars reticulata (Llorens et al., 1982). Recognized as a striatonigral pathway marker, CD10 also denotes a subset of striatal efferent fibers (Llorens et al., 1982). Barnes et al. have identified CD10 as the primary somatostatin-degrading enzyme in brain regions such as the striatum and hippocampus (Barnes et al., 1995). Further research reveals CD10’s enrichment in GABAergic neurons, with expression in GABAergic and metabotropic glutamate 2/3 receptor-positive neurons, but not in catecholaminergic or cholinergic neurons, suggesting specificity in cellular expression (Fukami et al., 2002). Its mRNA predominates in parvalbumin-expressing interneurons, while the protein localizes to parvalbuminergic synapses, crucial for soluble Aβ peptide degradation (Fukami et al., 2002; Madani et al., 2006; Hüttenrauch et al., 2015). As a versatile neuropeptidase, CD10 partakes in various brain functions, influencing pain response and aggression through its role in degrading enkephalins (Roques et al., 1993), and impacting the metabolism of sensory and inflammatory peptides, such as tachykinins and neurokinins (Turner and Nalivaeva, 2007). CD10 knockout models have linked the enzyme’s absence with hyperalgesia in visceral pain (Fischer et al., 2002), and have shown that its deficiency can alter locomotion and escalate aggression (Fischer et al., 2000; Chen et al., 2020).

Clinically, CD10’s regulation of the natriuretic peptide (NP) pathway in the heart positions it as a potential biomarker and therapeutic target for heart failure (HF) (Bayes-Genis et al., 2016; Arrigo et al., 2018). In the reproductive system, CD10’s involvement ranges from sperm formation to follicle maturation and ovarian function, mediated through its interaction with bioactive peptides like endothelin and AngII (Ghaddar et al., 2000; Zappulla and DesGroseillers, 2001; Subirán et al., 2010). Its activity variations throughout the estrous cycle, particularly during metestrus, indicate its role in ovarian function and the renin-angiotensin system (Pereira et al., 2009; Gonçalves et al., 2012; Pereira et al., 2020). Its expression in various placental and fetal membrane cell populations, with the ability to hydrolyze oxytocin, hints at a significant role in maternofetal interactions (Johnson et al., 1984; Imai et al., 1994). Its identity as skin fibroblast elastase and upregulation during human skin fibroblast senescence link CD10 to skin aging processes (Morisaki et al., 2010). Furthermore, CD10 is implicated in bone metabolism, with expression correlated with bone growth, particularly noted in newborns compared to adults (Ruchon et al., 2000).

## Role of CD10 in different types of diseases

The expression and regulatory pathways of CD10 have been increasingly recognized as crucial factors linked to the pathogenesis of numerous diseases, notably including various types of tumors, AD, cardiovascular diseases, obesity, diabetes and musculoskeletal diseases. The intricate role that CD10 plays in these conditions suggests that it could be a potential target for therapeutic intervention. In this section, we aim to delve into the correlations between CD10 expression levels and disease progression, examine the underlying molecular mechanisms that govern CD10’s regulatory functions, and explore how modulation of CD10 activity may influence disease outcomes.

### CD10 and hematologic tumors

CD10 plays a pivotal role in regulating the activation of immune cells through its capacity to degrade inflammatory peptides, including endothelin, bradykinin, atriopeptin, and interleukin-1. This enzymatic function is vital in the immune response and inflammation modulation (Delikat et al., 1994; Xie et al., 2011a; Xie et al., 2011b). B-lineage ALL is the most common pediatric malignancy. Studies have indicated that the presence of CD10 expression on blast cells correlates with several favorable presenting features in B-ALL. Notably, a lack of CD10 expression in B-ALL patients is associated with a poor prognosis, and its absence may similarly signal an adverse prognosis in T-ALL (Vaughan et al., 1988). Research by Dakka et al. has demonstrated that the co-expression of CD34 and CD10 is indicative of a significantly better prognosis in patients with B-lineage ALL (Dakka et al., 2009). In the context of B-ALL diagnosis, CD10 serves as a critical antigen delineating the developmental stages of lymphoid cells: CD10 is not expressed in pro-B ALL patients, but is present in all patients with common B-ALL. Moreover, the majority of pre-B ALL patients who test positive for cytoplasmic IgM also express CD10 (Tomonaga, 2009). CD10’s diagnostic relevance is not limited to B-ALL; it is also a critical biomarker for subclassifying various forms of acute leukemias and aiding in the identification of non-Hodgkin lymphomas. This highlights its broad applicability in hematological evaluations and its potential impact on disease management and outcome predictions (Sánchez-Céspedes et al., 2013).

### CD10 and nonhematologic tumors

Aberrant expression of CD10 is closely linked to the progression and enhanced malignancy of various nonhematologic tumors. A range of studies, from those conducted by Ogawa et al., in 2002 to more recent research by Jiang et al., in 2021, have consistently underscored this association between CD10 expression and tumor progression (Ogawa et al., 2002; Bilalovic et al., 2004; Braham et al., 2006; Kadota et al., 2015; Jiang et al., 2021). Despite these findings, the precise biological role and mechanisms of CD10 within these processes remain elusive. Emerging hypotheses suggest that CD10 may influence tumor aggressiveness through its role in extracellular matrix degradation and the modulation of intracellular signaling pathways, as noted by researchers such Biasoli et al. (2005), Kim et al. (2010), Khanh do et al. (2011). While certain studies posit that CD10 overexpression is a standalone negative prognostic indicator in cancer prognosis, as supported by findings from Ahem et al. and others (Avery et al., 2000; Ordi et al., 2003; Jung and Kuo, 2005; Ahlem et al., 2015; Gjorgova-Gjeorgjievski et al., 2021), there is contrasting evidence indicating downregulation of CD10 in various cancers, including lung and prostate cancer, as reported by Papandreou et al. (1998), Kristiansen et al. (2002).

CD10 is expressed on normal prostate epithelium and plays a critical role in mitigating the proliferative and survival signals in prostate cells by inactivating neuropeptides such as endothelin-1 (ET-1) and bombesin. These neuropeptides are known to induce phosphorylation of insulin-like growth factor-1 receptor (IGF-IRβ) and protein kinase B (Akt). Sumitomo et al. has demonstrated that restoring CD10 expression in CD10-negative PC cells can inhibit the ET-1-mediated phosphorylation of IGF-IRβ and Akt, consequently blocking ET-1’s protective effects against apoptosis under serum-deprived conditions (Sumitomo et al., 2001). Further studies by Zheng et al. have elucidated that CD10 may impede the bombesin-induced activation of RhoA, a key regulator of cell migration and invasion in PC cells (Zheng et al., 2006). Interestingly, according to Ho et al., expression of CD10 by PC correlates with poor disease outcome and lower rates of patient survival (Ho et al., 2013). Dall'Era et al., also demonstrated that CD10-positive PC cells are often present in lymph node metastases; they also found that CD10 expression correlates with a more aggressive and potentially malignant phenotype of PC (Dall Era et al., 2007; Freedland et al., 2003). Furthermore, CD10 is believed to function as an immune receptor within the cellular membrane of PC cells, forming complexes with PTEN that influence cell migration, proliferation, and survival through the signaling pathways of focal adhesion kinase (FAK) and PTEN/AKT (Sumitomo et al., 2004). Interestingly, CD10 DNA hypermethylation has been discovered in some PC patients who exhibit a loss of CD10 expression, indicating that epigenetic mechanisms could play a pivotal role in the silencing of CD10 (Osman et al., 2004).

CD10 is normally expressed in the small intestine but is typically absent in the colon. Nevertheless, it is found to be aberrantly expressed in a minority of colorectal cancer (CRC) cases, where its presence is linked with tumor metastasis and an unfavorable prognosis. Research by Raposo et al. suggests a dynamic role for CD10 across the stages of colorectal cancer development: it may act to restrain cell motility in the early stages while later contributing to enhanced cell survival (Raposo et al., 2018). Several studies have established a significant association between CD10 expression and the increased occurrence of liver metastases in advanced CRC (Yao et al., 2002; Fujimoto et al., 2005; Kumagai et al., 2015). Investigations into the molecular mechanisms reveal that CD10 may facilitate the spread of CRC to the liver by counteracting the anti-tumor properties of hepatic methionine-enkephalin (MENK) (Kuniyasu et al., 2010). The application of CD10 inhibitors has been shown to suppress CRC cell proliferation by inducing cell cycle arrest in the G0/G1 phase, stimulating ERK1/2, and decreasing the phosphorylation of mTOR, 4E-BP1, and p70S6K. However, these inhibitors do not trigger apoptosis in CRC cells (Mizerska-Kowalska et al., 2019). Further studies by Mizerska-Kowalska et al. point to the involvement of CD10 in CRC cell growth, proliferation, migration, and invasiveness, which it mediates by regulating cancer cell motility via the Akt/FAK signaling pathways (Mizerska-Kowalska et al., 2016). They also discovered that CD10 expression is vital for the synthesis of TGF-β1, and its inhibition corresponds with decreased levels of TGF-β1-a tumor suppressor known to curb cell proliferation and induce apoptosis through the upregulation of cell cycle inhibitors (like p21) and the activation of MAP kinases and PI3K/Akt pathways (Mizerska-Kowalska et al., 2021).

The overexpression of CD10 has been identified as a valuable diagnostic marker that can differentiate between benign and malignant phyllodes tumors in the breast (Ibrahim, 2011; Tariq et al., 2015; Puri et al., 2016), as well as distinguish invasive breast carcinoma from ductal carcinoma in myoepithelial cells of the breast (Kalof et al., 2004). The presence of CD10 in the stromal cells of invasive breast cancer is indicative of a poor prognosis, being associated with reduced survival rates and increased invasive and metastatic capabilities (Kalof et al., 2004; Toussaint et al., 2010; Bacha et al., 2020). Additionally, significant correlations have been drawn between CD10 and clinical indicators of aggressive and invasive breast cancer behaviors, including higher histological grades and the presence of nodal metastasis (Louhichi et al., 2018). Emerging studies suggest that CD10 plays a critical role in modulating tumor expansion and spread by influencing the tumor microenvironment, which encompasses adjacent support cells and extracellular matrix components. The expression of CD10 within the tumor microenvironment, potentially induced by breast tumors with a cancer stem cell phenotype, may facilitate the breakdown of the extracellular matrix, thereby enabling neoplastic cells to infiltrate the lymphatic system and form distant metastases (Gattazzo et al., 2014). Louhichi et al. have identified a significant link between stromal CD10 expression and the breast cancer stem cell phenotype (Louhichi et al., 2018). Desmedt et al. have emphasized the prognostic significance of CD10-positive stromal cells in breast cancer, particularly in relation to HER2-positive subtypes. They observed notable shifts in the presence of CD10-positive stromal cells during breast cancer progression and recognized their role in remodeling the tumor matrix (Desmedt et al., 2012). Moreover, Su et al. proposed that CD10+GPR77+ cancer-associated fibroblasts (CAFs), a type of stromal cell in the tumor microenvironment, contribute to the secretion of interleukin (IL)-6/8 and create a survival niche that not only nurtures the stemness of breast cancer cells but also shields them from the lethal effects of chemotherapy (Su et al., 2018).

CD10 expression has also been observed to affect patient outcomes in other forms of cancer. Specifically, a study by Li et al. reported that higher CD10 expression in head and neck squamous cell carcinoma (HNSCC) correlates with worse overall survival (OS) and recurrence-free survival (RFS). The study further reveals a correlation between CD10 expression and increased IL-6/IL-8-mediated M1 macrophage activity, which may explain the poor prognosis observed in HNSCC cases (Li et al., 2021). In esophageal squamous cell carcinoma (ESCC), research by Lee et al. has revealed that 30% of the examined ESCC samples exhibited CD10 expression in cancer cells to varying degrees. Their findings suggest that the transcription factor Twist1 is capable of binding to the CD10 promoter region, thereby upregulating CD10 transcription within ESCC cells. Moreover, they discovered that silencing CD10 could impede the growth of ESCC xenografts in nude mice (Lee et al., 2015). These data add to the growing body of evidence that CD10’s regulatory function on cell proliferation and survival extends across different cancer types, potentially offering a common thread in the search for targeted cancer therapies.

### CD10 and AD

The accumulation of amyloid-beta peptide (Aβ) in the brain is a hallmark of AD, leading to synaptic dysfunction, neuronal loss, and impaired memory function (Hardy and Selkoe, 2002; Turner et al., 2004; Murphy et al., 2007; Miners et al., 2008). CD10 is crucial for degrading Aβ and eliminating it from the brain (Maruyama et al., 2005). As a primary Aβ-degrading enzyme, CD10 is downregulated in the early stages of AD, corresponding with increased Aβ accumulation in the brain and the formation of amyloid-like structures (Yasojima et al., 2001; Nalivaevaa et al., 2004; Madani et al., 2006; El-Amouri et al., 2008). Decreased CD10 levels are associated with AD-like pathology and behavioral deficits. For instance, Dubrovskaia et al. found that injections of phosphoramidon or thiorphan (CD10 inhibitors) into the sensorimotor cortex of rats led to a decline in spatial memory and disrupted neuronal networks, impairing the cognitive functions of the animals (Nalivaeva et al., 2008; Dubrovskaia et al., 2009).

Elevating CD10 levels and restoring its ability to degrade amyloid offers a promising strategy to potentially slow or even reverse AD progression. Yasojima et al. observed that in AD patients, CD10 levels were the lowest in regions like the hippocampus (HPC) and temporal gyrus-areas vulnerable to senile plaque development. In stark contrast, the caudate and peripheral organs, resistant to senile plaques, exhibited the highest levels (Yasojima et al., 2001). The quest now is to discover methods to enhance CD10 levels in the brain to mitigate Aβ buildup. However, the blood-brain barrier poses a challenge, making it unlikely for peripheral CD10 to access the brain via blood circulation (Spencer et al., 2014). Leissring et al. showed that neurons overexpressing CD10 transgenically saw a significant drop in brain Aβ levels, stalling or entirely preventing amyloid plaque formation and related cytopathology while also rescuing the premature death seen in amyloid precursor protein transgenic mice (Leissring et al., 2003). Another study by Liu et al. indicated that a plasma-secreted soluble form of CD10 could efficiently reduce the Aβ burden in a transgenic AD mouse model (Liu et al., 2010). Marr et al. reported that a unilateral intracerebral injection of a lentiviral vector expressing human CD10 halved Aβ deposits compared to the untreated side and also mitigated neurodegenerative changes in the transgenic mice’s frontal cortex and hippocampus (Marr et al., 2003). A unique observation by Kobayashi et al. highlighted that exosomal CD10 from the masseter muscle could travel to the HPC during mastication via the trigeminal nerve and intracerebral axonal pathways. This process implies that chewing might elevate CD10 levels in the HPC and other brain regions, aiding in Aβ degradation. This transport mechanism could pave the way for innovative drug delivery methods to the brain, such as intramuscular injections into the masseter (Kobayashi et al., 2019). Liu et al. demonstrated that Sodium Tanshinone IIA Sulfonate (STS) could counteract Aβ1-42-induced apoptosis, minimize oxidative damage, and alleviate ER stress in HT22 cells, likely by amplifying CD10 expression to degrade Aβ. As such, introducing STS as a preventive measure might complement therapeutic strategies for AD (Liu et al., 2020).

### CD10 and cardiovascular diseases

CD10 degrades a variety of vasoactive peptides, including vasodilators such as natriuretic peptides, bradykinin, and adrenomedullin, as well as vasoconstrictors like angiotensin II and endothelin-1. Given these roles, inhibiting CD10 presents a promising therapeutic approach for cardiovascular diseases related to hypertension and fluid overload. Notably, the atrial natriuretic peptide (ANP) not only acts as a potent vasodilator and natriuretic agent but also inhibits the renin-angiotensin system (RAS) by reducing renin and aldosterone release. Bayés-Genís et al. suggests that inhibiting CD10 can enhance the effects of naturally occurring natriuretic peptides (NPs). These peptides help promote natriuresis, induce vasodilation, and decrease cardiac hypertrophy and fibrosis in HF patients (Bayés-Genís et al., 2015). Another study by Arrigo et al. emphasized the importance of the NP pathway in HF, and indicated that soluble CD10 can degrade NP, offering a potential treatment strategy for HF (Arrigo et al., 2018). Arrigo et al. have shown an inverse relationship between soluble CD10 levels and both left and right ventricular function in patients with hypertrophic cardiomyopathy (Arrigo et al., 2018; Yoshihisa et al., 2019a). Yoshihisa et al. found that circulating soluble CD10 might predict prognosis in HF patients with reduced ejection fraction (HFrEF) by degrading vasodilator peptides such as NPs (Yoshihisa et al., 2019b). Additionally, both Prausmüller et al. and Pavo et al. have demonstrated that the expression levels of membrane CD10 in neutrophils and granulocytes, respectively, are correlated with heart failure prognosis, with lower expression linked to increased severity and higher expression associated with better outcomes (Pavo et al., 2019; Prausmüller et al., 2021).

### CD10 and obesity and diabetes

Recent studies have shed light on CD10’s crucial role in the onset of obesity and type 2 diabetes. Gul et al. have associated elevated CD10 levels with insulin resistance, pointing to its connection with endocrine and metabolic disorders (Gul et al., 2022). Supporting this notion, Kim et al. elaborated on CD10’s ability to promote adipogenesis by activating the insulin-mediated PI3K-Akt signaling pathway (Kim et al., 2017). Furthermore, Standeven et al. observed increased CD10 production in human adipocytes during differentiation, while also noting raised CD10 levels in obese, insulin-resistant mice (Standeven et al., 2011). Interestingly, Muenzner et al. reported that green tea can notably decrease body fat and prevent its further accumulation in Berlin fat mice. This effect is attributed to the enhanced expression and activity of CD10 in peripheral areas, such as the kidney and intestine, leading to the downregulation of orexigens. This discovery paves the way for a new potential treatment approach for obesity (Muenzner et al., 2016).

CD10’s impact on glucose homeostasis is profound. This enzyme hydrolyses peptides essential to glucose metabolism, potentially leading to impaired glucose homeostasis-a distinctive trait of type 2 diabetes (T2D). Parilla et al. explored this relationship further and revealed that under conditions associated with T2D, upregulated CD10 can disrupt glucose balance. However, when CD10 activity is deficient or inhibited, there’s improved glucose tolerance, insulin sensitivity, and enhanced pancreatic *β*-cell function (Parilla et al., 2018). Becker et al. contributed to this discussion, noting that CD10-deficient mice, even on a normocaloric diet, started to display obesity traits from 6–7 months of age. By 1 year, these mice showed significant spikes in fasting blood glucose and a decreased ability to manage an oral glucose load (Becker et al., 2010). Standeven et al. observed a direct correlation between CD10 activity, body mass index (BMI), and insulin resistance, especially in subjects with multiple cardiovascular risk factors (Standeven et al., 2011).

The expression and activity of CD10 are associated with various complications of diabetes. Exploring the potential linkage between diabetes and AD, Morales-Corraliza et al. observed decreased CD10 levels in the hippocampus of insulin-controlled diabetes mellitus monkeys, which corresponds with an increase in localized Aβ (Morales-Corraliza et al., 2016). Urinary CD10 levels are increased in patients with diabetes and could be used as early biomarkers to predict the incidence or progression of chronic kidney disease (CKD) at early stages among individuals with type 2 diabetes (Gutta et al., 2018). CD10 expression and activity increasing wounds and skin in type 2 diabetes mellitus, which reducing substance P and diminishing normal response to injury, and then cause delayed wound healing (Muangman et al., 2003).

### CD10 and inflammatory arthritis

The relationship between CD10 and inflammation has been extensively elucidated by Solan et al. and Shipp et al. (Shipp et al., 1991b; Solan et al., 1998). Substance P (SP) is a small peptide produced by nociceptive nerve fibers. It transmits pain signals, induces vasodilation and edema in peripheral vessels, and promotes the migration of immune cells from circulation into the surrounding tissue (Clockaerts et al., 2010). Through enzymatic catabolism, CD10 effectively modulates the biological activity and degradation of SP, thus exerting an anti-inflammatory function. SP plays a role in the development of inflammatory arthritis (Proud and Kaplan, 1988; Konttinen et al., 1994) and is found at significantly higher levels in the synovial fluids of individuals with rheumatoid arthritis and gout (Marshall et al., 1990). Researchers have long discovered that various cells isolated from joint sites, such as synovial fibroblast-like cells (Solan et al., 1998), synovial mesenchymal stem cells (MSCs) (Denkovskij et al., 2015), mesenchymal progenitor cells (MPCs) (Jones et al., 2004) and infrapatellar fat pad (IFP)-MSCs (Kouroupis et al., 2019) (some of which may be the same type of cells with different names), are capable of expressing CD10. Studies have attempted to utilize MPCs or MSCs for the treatment of arthritis and inflammation-induced joint injuries (such as articular cartilage damage) (Kurose et al., 2010), and have demonstrated that the therapeutic efficacy of MPCs and MSCs may be associated with the high expression of CD10. One of the more representative works in these studies comes from Correa and his team. In their series of studies, Correa et al. extensively elucidated their efforts to utilize IFP-MSCs-based therapeutics in addressing the adverse effects of immune-mediated inflammatory joint alterations associated with conditions like osteoarthritis. They also provided detailed molecular mechanisms of how IFP-MSCs effectively inhibit the arthritis inflammation, including the regulation of inflammatory factor expression, the modulation of inflammatory pathways activation, and the induction of SP degradation through the overexpression of CD10 (Kouroupis et al., 2019; Greif et al., 2020; Kouroupis et al., 2020; Kouroupis et al., 2023). Regarding the relevant characteristics of IFP-MSCs overexpressing CD10, we will further discuss them in the ‘CD10 AND STEM CELLS' section.

## CD10 and stem cells

CD10 is commonly expressed in stem cells and progenitor cells, and is widely recognized as a pivotal marker for these cells (Bühring et al., 2007; Poblet and Jiménez, 2008; Bachelard-Cascales et al., 2010). There’s been a longstanding uncertainty about the specific requirements and differentiation markers for human B lymphopoiesis. A study by Ichii et al. addressed this ambiguity by assessing the differentiation potential of lymphoid progenitors (CLPs) from various sources-umbilical cord blood, adult bone marrow, and granulocyte colony-stimulating factor (G-CSF) mobilized peripheral blood-based on CD10 density. Their findings indicate that an increase in CD10 expression aligns with the differentiation potential and developmental stage of B lymphocytes in human B lymphopoiesis (Ichii et al., 2010). Bachelard-Cascales et al. identified CD10 as a key regulator in mammary ductal development, distinguishing progenitors from luminal cells when combined with epithelial cell adhesion molecule (EpCAM). They found CD10-high EpCAM-low cells are enriched with progenitors and suggested that CD10 and beta1-integrin interactions are essential for maintaining the mammary lineage’s progenitor and stem cell pools (Bachelard-Cascales et al., 2010). Ding et al. identified that a subset of human adventitial cells, which ensheath arteries and veins, express the CD10/CALLA metalloprotease. While both CD10^+^ and CD10-adventitial cells exhibit mesenchymal stem cell characteristics, the CD10^+^ subset showed enhanced proliferation, clonogenicity, and osteogenic potential, suggesting its role in perivascular MSC function and cell fate determination. Their findings indicate a potential role for CD10^+^ perivascular cells in vascular remodeling and calcification (Ding et al., 2020).

In addition, effective stem cell therapy is dependent on the stem cell quality that is determined by their differentiation potential, impairment of which leads to poor engraftment and survival into the target cells. However, limitations in our understanding and the lack of reliable markers that can predict their maturation efficacies have hindered the development of stem cells as an effective therapeutic strategy. In a study investigating adipose-derived stem cells (ASCs) from subcutaneous and visceral white adipose tissue, Ong et al. found that CD10 was predominantly expressed in subcutaneous ASCs. Notably, cells with higher CD10 expression demonstrated superior differentiation capabilities. This suggests that CD10 can serve as a valuable marker for tracking and differentiating subcutaneous stem cell populations (Ong et al., 2014). Chakraborty et al. identified CD10 plays a pivotal role in lipid accumulation during adipogenesis. Their findings reveal that CD10 orchestrates a unique, non-canonical pathway reliant on the peroxisome proliferator-activated receptor gamma (PPARγ) for adipogenic differentiation. This pathway is crucial for the maturation of ASCs into high-quality white adipose tissue and its subsequent browning (Tran et al., 2008; Tran and Kahn, 2010; Chakraborty et al., 2021). Kouroupis et al. investigated the properties of human infrapatellar fat pad (IFP)-MSCs under different culture conditions. They found that IFP-MSCs processed with human platelet lysate (hPL) or chemically reinforced medium (Ch-R) exhibited a CD10-high phenotype and displayed enhanced proliferation, differentiation, and immunomodulatory profiles. Notably, these CD10-rich IFP-MSCs effectively reversed signs of synovitis and IFP fibrosis *in vivo*. (Kouroupis et al., 2019; Kouroupis et al., 2020).

Cancer stem cells (CSCs) are characterized by their ability to initiate tumors and self-renew. It has been well-documented that CSCs play a pivotal role in conferring therapeutic resistance and contributing to the failure of cancer treatments (Bao et al., 2006; Li et al., 2008). Elevated CD10 expression has been significantly linked with the upregulation of genes associated with CSCs, including *CD44*, *ALDH1*, *BMI1*, *NANOG*, *OCT4* and *SOX2* (Xia, 2014; Lazarevic et al., 2018). In oral cancer, heightened CD10 levels are implicated in fostering CSC-like traits, promoting the expression of key CSC-related genes such as *BMI1*, *OCT4*, and *SOX2*, and amplifying capabilities crucial to malignancy progression like cell migration, invasion, spheroid genesis, and resistance to chemotherapy (Pu et al., 2021). Furthermore, Wang et al. observed that oral squamous cell carcinoma (OSCC) cells with high CD10 expression exhibit pronounced CSC-like properties, including enhanced self-renewal, cell cycle modulation, and elevated expression of epithelial mesenchymal transition (EMT)-related CSC markers like *N-Cadherin*, *Vimentin*, and *Slug*, alongside reduced levels of the epithelial marker E-Cadherin. These cells also demonstrate increased tumor growth, EMT progression, and resistance to cisplatin (Wang et al., 2021). Fukusumi et al. identified CD10 as a crucial factor associated with therapeutic resistance and cancer stem cell-like properties in head and neck squamous cell carcinoma (HNSCC). They treated HNSCC cell lines with cisplatin or radiation and observed upregulation of CD10, along with other cell surface antigens, in response to treatment. Notably, the cisplatin-resistant cell line exhibited prominent CD10 upregulation. Further isolation of CD10-positive subpopulations demonstrated their increased resistance to cisplatin, fluorouracil, and radiation, as well as enhanced *in vitro* sphere formation and *in vivo* tumor formation capacity. Additionally, the CD10-positive subpopulation expressed higher levels of the CSC marker OCT3/4. These findings suggest that CD10 may serve as a potential target molecule for addressing refractory HNSCC, highlighting its significance in treatment resistance and CSC-related characteristics (Fukusumi et al., 2014).

## CD10 and COVID-19

Coronavirus-disease-2019 (COVID-19) has emerged as a global pandemic, caused by the widespread severe-acute-respiratory-syndrome-coronavirus-2 (SARS-CoV-2). While the World Health Organization (WHO) has declared the global COVID-19 pandemic over, the health impacts of COVID-19 infections, its complications, and lingering effects are far from eradicated. The cellular and molecular biological mechanisms of the diseases triggered by the virus remain to be elucidated. In the early stages of the COVID-19 pandemic, there were no specific vaccines or effective therapeutic interventions. A number of discussions have highlighted the potential involvement of CD10 in the complications arising after the viral infection. Vassallo et al. (Vassallo et al., 2020) observed that in a group of elderly patients infected with COVID-19, the levels of CD10^+^ B cells were inversely correlated with the severity of clinical symptoms. Spijkerman et al. (Spijkerman et al., 2021) discovered that in all COVID-19 patients, regardless of the severity of the disease, the expression of CD10 in neutrophils was very low. Mohammed et al. (Mohammed El Tabaa and Mohammed El Tabaa, 2020) elaborated in their article the detailed molecular mechanisms and relevant pathways of CD10 as a potential therapeutic target for COVID-19. Firstly, CD10 can mitigate COVID-19-induced pulmonary inflammation, particularly neutrophil infiltration, thereby preventing lung damage and reducing pulmonary fibrosis. This includes modulating the chemotactic responsiveness via cleaving the chemotactic peptide formyl-Met-Leu-Phe (fMLP) to prevent neutrophil infiltration, decreasing the pro-inflammatory, oxidative, and pro-fibrotic effects by regulating the Cathepsin G/Ang I/Ang II system and Ang (Maguer-Satta et al., 2011; Nalivaeva et al., 2020; Greif et al., 2020; DElia et al., 2017; Feygina et al., 2019; DAdamio et al., 1989; McKerrow, 1987)/TGF-β system (Manolis et al., 2020), as well as breaking down bradykinin to inhibit its role in activating and recruiting inflammatory cells. Additionally, CD10 may prove beneficial in managing individuals at high risk for COVID-19 who face various obstacles in their management, such as cardiovascular and hypertensive patients, diabetic patients, and elderly individuals with reduced androgen or estrogen levels.

While multiple sets of data confirm CD10’s anticipated role in alleviating pulmonary inflammation, its potential to reduce exacerbations of acute severe pneumonia in COVID-19 patients has not yet been emphasized. With the conclusion of the pandemic, public interest in COVID-19 has significantly diminished. In China, COVID-19 has been categorized as a Class B infectious disease for management (Luo et al., 2023). However, whether we should prepare in advance for the occurrence of the next outbreak? This is a question that all researchers need to contemplate.

## CD10 as a therapeutic target

As a pervasive distributed endopeptidase, CD10 plays a pivotal role in the onset and progression of various diseases. Treatment attempts targeting CD10 have been mentioned multiple times in the previous sections. One of the more typical applications involves increasing the levels of CD10 within tissues to treat diseases caused by Aβ accumulation, such as AD (Nalivaeva and Turner, 2019) (refer to Section 2.3) and retinal diseases. As Aβ is believed to have detrimental effects on posterior eye tissues and is implicated in retinal diseases like age-related macular degeneration, Parthasarathy et al. reported in their study that in a mouse model through the intra-vitreal delivery of sNEP (recombinant soluble form of CD10), decreases in ocular Aβ were induced while maintaining good electroretinographic responsiveness in a controlled, dose-dependent manner (Parthasarathy et al., 2015).

In comparison to CD10, what is closer to clinical application are multiple CD10 inhibitors. By targeting this enzyme with inhibitors, it has paved the way for the creation of innovative drugs and therapies, holding the potential to shift the current paradigm in the management of multiple diseases. After nearly three decades of continuous exploration, several CD10 inhibitors have been developed, with some having passed clinical trials and commenced commercial sales. The most successful application of CD10 inhibitors has been in the management of HF. Candoxatril (candoxatrilat) was one of the earliest CD10 inhibitors used in the treatment of chronic HF, which was demonstrated to exhibit vasodilatory activity at certain doses (Kentsch and Otter, 1999; McDowell and Nicholls, 1999). Owing to the limitations of using a single drug and its adverse effects, there’s a growing interest in dual drug research. Sacubitril/valsartan, a pioneering drug in the class of dual CD10 and angiotensin receptor inhibitors (ARNI), has been proven to enhance outcomes for patients with symptomatic HF featuring reduced systolic function, and its use for the treatment of HF was approved by the FDA in 2015 (McMurray et al., 2014; Chen and Burnett, 2017; Khder et al., 2017; Kario, 2018; Pascual-Figal et al., 2021). In a study conducted by Seferovic et al., the efficacy of sacubitril/valsartan in treating patients with HFrEF and coexisting diabetes was investigated. The results highlighted that those administered with sacubitril/valsartan saw a more pronounced decrease in HbA1c levels-a key indicator of long-term glucose control-compared to counterparts treated with enalapril (Seferovic et al., 2017). A key mechanism of sacubitril/valsartan is its ability to increase nitric oxide bioavailability, mitigate systemic oxidative stress, apoptosis, and hypertrophy, thereby diminishing infarction size and slowing post-acute myocardial infarction cardiac remodeling (Trivedi et al., 2018).

CD10 inhibitors are increasingly being recognized for their potential in wound healing therapies. Following cutaneous injury, CD10 plays a role in degrading the released substance P. Interestingly, heightened CD10 activity is observed in chronic wounds and in the skin of diabetic patients. A study by Spenny et al. highlighted that diabetic mice treated with the CD10 inhibitor, thiorphan, experienced faster wound closure compared to those treated with saline. Similarly, research from Genova et al. found that CD10-deficient mice exhibited accelerated corneal wound healing post alkali burn injury compared to the wild type mice (Spenny et al., 2002). Moreover, the introduction of thiorphan to wild type mice showed a notable boost in the post-injury healing rate (Genova et al., 2018), which may be provide a novel therapeutic option for patients with corneal injury.

CD10 inhibitors have also shown potential application value in the field of reproductive health. For instance, the CD10 inhibitor UK-414,445 has been shown to enhance genital blood flow in response to pelvic nerve stimulation in female rabbits without notably impacting blood pressure. This offers a potential new pharmacological solution for treating female sexual arousal disorder (Angulo, 2010). The opiorphin peptide family, potent CD10 inhibitors, are also gaining attention in erectile physiology. Davies et al. identified genes encoding opiorphins as potential markers for erectile dysfunction (ED). Furthermore, intracorporal injection of sialorphin, an opiorphin family peptide, can improve erectile function (Davies, 2009). Several studies indicate that CD10 has considerable negative effects on sperm motility. Intriguingly, a number of studies have shown that opiorphin, found in human seminal plasma, can enhance sperm’s progressive motility. Furthermore, opiorphin has been observed to positively influence sperm motility parameters, especially in cases of male infertility marked by asthenozoospermia, leading to improved results in intrauterine insemination (Subirán et al., 2008; Pinto et al., 2010; Bosler et al., 2014; Fritz et al., 2019).

Another important application of CD10 inhibitors is in pain management, such as cancer pain (Menéndez et al., 2008; Gonzalez-Rodriguez et al., 2017). Inhibiting the function of CD10 with a CD10 inhibitor can increase the concentration of endogenous pain-relieving substances (such as enkephalins) in the body, thereby achieving analgesic and anti-allergic effects (Thanawala et al., 2008). However, as mentioned earlier, CD10 can also reduce pain caused by inflammation by degrading SP (refer to Section 2.6). Therefore, the role of CD10 in these two aspects of pain management seems contradictory. This illustrates that CD10 can have different effects in different types of pain and inflammation, depending on its molecular targets and the local pathological processes involved. In order to unlock their potential applications, research on CD10 inhibitors is still evolving and being actively explored, and some progress has been made in laboratory studies. For instance, CD10 inhibitors have been used for the modulation or treatment of conditions such as cancer development (Mizerska-Dudka and Kandefer-Szerszeń, 2015), diabetes (diabetes complications) (Packer and Kitzman, 2018; Esser and Zraika, 2019), stress response, addiction, and food intake (Roques, 2018; Ramírez-Sánchez et al., 2019). However, most of these studies have not yet reached the clinical trial phase, and no commercial drugs have been marketed.

## Conclusion and perspectives

As mentioned earlier, CD10 has been proven to be an important regulatory factor in various diseases. With the advancement of medical research, our understanding of CD10 has become increasingly comprehensive. It not only plays a key role in cell proliferation, differentiation, and migration but is also associated with multiple signaling pathways. This gives us a compelling reason to further explore the potential of CD10 as a therapeutic target. As scientific technology progresses and research continues to deepen, CD10 will undoubtedly occupy an increasingly important position in the therapeutic field.

However, we must also recognize that in stark contrast to the bustling laboratory research, clinical studies on CD10 and its inhibitors are exceedingly rare. Moreover, because the molecular mechanisms are still unclear, drug development and translational outcomes for targeting CD10 are meager, especially for obesity, aging, cancer, and AD, with no successful drugs having reached the marketplace. Another point of note is that studies in animal models indicate that CD10 inhibition might result in adverse consequences such as angioedema, bronchial reactivity, inflammation, cancer, and could predispose to polyneuropathy and AD (Campbell, 2017). Therefore, the side effects of CD10 inhibitors must be taken seriously, and more animal studies and clinical trials are needed to verify the safety and efficacy of the potential applications of CD10 inhibitors.

In summary, in this article, we have reviewed several research papers on CD10 published in recent years, summarizing the biological functions of CD10, its expression in tissues and organs, its relevance to the development of various diseases, and its regulatory molecular mechanisms. We have also summarized the applications of CD10 inhibitors in disease treatment. Our goal is to spark further interest among researchers in the study of CD10 and its inhibitors, thereby promoting the application of CD10 and its inhibitors in disease prediction, diagnosis, and treatment.
